# Effects of eye movement desensitization and reprocessing (EMDR) on non-specific chronic back pain: a randomized controlled trial with additional exploration of the underlying mechanisms

**DOI:** 10.1186/1471-2474-14-256

**Published:** 2013-08-30

**Authors:** Jonas Tesarz, Andreas Gerhardt, Sabine Leisner, Susanne Janke, Mechthild Hartmann, Günther H Seidler, Wolfgang Eich

**Affiliations:** 1 Department of Internal Medicine and Psychosomatics, University Hospital Heidelberg, Im Neuenheimer Feld 410, Heidelberg D-69120, Germany; 2 Department of Internal Medicine and Psychosomatics, University Hospital Heidelberg, Thibautstr. 2, Heidelberg D-69115, Germany

**Keywords:** Eye movement desensitization and reprocessing, EMDR, Non-specific chronic back pain, Psychological trauma, Randomized controlled trial, Study protocol

## Abstract

**Background:**

Non-specific chronic back pain (CBP) is often accompanied by psychological trauma, but treatment for this associated condition is often insufficient.

Nevertheless, despite the common co-occurrence of pain and psychological trauma, a specific trauma-focused approach for treating CBP has been neglected to date. Accordingly, eye movement desensitization and reprocessing (EMDR), originally developed as a treatment approach for posttraumatic stress disorders, is a promising approach for treating CBP in patients who have experienced psychological trauma.

Thus, the aim of this study is to determine whether a standardized, short-term EMDR intervention added to treatment as usual (TAU) reduces pain intensity in CBP patients with psychological trauma vs. TAU alone.

**Methods/design:**

The study will recruit 40 non-specific CBP patients who have experienced psychological trauma. After a baseline assessment, the patients will be randomized to either an intervention group (n = 20) or a control group (n = 20). Individuals in the EMDR group will receive ten 90-minute sessions of EMDR fortnightly in addition to TAU. The control group will receive TAU alone. The post-treatment assessments will take place two weeks after the last EMDR session and six months later.

The primary outcome will be the change in the intensity of CBP within the last four weeks (numeric rating scale 0–10) from the pre-treatment assessment to the post-treatment assessment two weeks after the completion of treatment.

In addition, the patients will undergo a thorough assessment of the change in the experience of pain, disability, trauma-associated distress, mental co-morbidities, resilience, and quality of life to explore distinct treatment effects. To explore the mechanisms of action that are involved, changes in pain perception and pain processing (quantitative sensory testing, conditioned pain modulation) will also be assessed.

The statistical analysis of the primary outcome will be performed on an intention-to-treat basis. The secondary outcomes will be analyzed in an explorative, descriptive manner.

**Discussion:**

This study adapts the standard EMDR treatment for traumatized patients to patients with CBP who have experienced psychological trauma. This specific, mechanism-based approach might benefit patients.

**Trial registration:**

This trial has been registered with ClinicalTrials.gov (NCT01850875).

## Background

Chronic pain conditions of the musculoskeletal system are common and have high socioeconomic relevance [1-3]. This is especially true for pain conditions with widely unknown pathogenesis, such as non-specific chronic back pain (CBP). In addition prevalence as well as the demand for consultation and treatment increases [4,5], thus causing high direct and indirect costs [1-3,6]. Despite high prevalence, CBP remains poorly understood and predominantly unspecific and inadequately treated.

Further, CBP is often complicated by the presence of mental co-morbidities, but treatment for this association remains insufficient. An important comorbidity that has been neglected so far is the role of psychological trauma in the development and maintenance of CBP. Traumatic events have higher prevalence rates in CBP patients as compared to pain-free controls or other diseases [7,8].

Between 10% and 50% of patients receiving tertiary care treatment for chronic pain have symptoms according to psychological trauma and posttraumatic stress disorder [9]. Moreover, chronic pain is associated with higher exposure to psychological trauma [10,11]. Contrary, only 23.8% of the German population reports a traumatic experience (fulfilling DSM-IV A criteria) and only 2.9% have diagnoses of a posttraumatic stress disorder [12]. Concerning traumatic experiences it was suggested that multiple traumas have a cumulative effect on physical health, including back pain and that the impact of the trauma on health may be independent of the development of a posttraumatic stress disorder [13]. This was supported in a prospective study that suggested that in the transition from acute to chronic pain cumulative trauma exposure appeared to be an independent risk factor [14]. In addition, absence of recent adverse life events independently predicted musculoskeletal health in a prospective study [15].

Considering the association of chronic pain and symptoms of posttraumatic stress disorder a model of shared vulnerability and mutual maintenance is discussed. The model purposes that physiological, affective, and behavioral components of psychological traumatization maintain and exacerbate symptoms of pain and, similarly, that cognitive, affective and behavioral components of chronic musculoskeletal pain maintain or exacerbate symptoms of posttraumatic stress disorder. Some of these factors are expected to constitute a shared vulnerability [9,16,17]. The model of mutual maintenance was partially confirmed in a prospective study by Jenewein et al. They reported a mutual maintenance of posttraumatic stress symptoms and pain intensity within the first six months, but, at 12-month follow-up pain intensity was significantly influenced by posttraumatic stress symptom, whereas such symptoms of traumatization were no longer influenced by pain intensity [18].

Concerning treatment of CBP [19-24] or psychological trauma [25-28] there are many guidelines. In spite of the high comorbidity of psychological trauma and pain, none of the guidelines for neither chronic pain nor psychological trauma incorporates the other, except the Expert Consensus Guideline Series for treatment of posttraumatic stress disorder [29]. Surprisingly, this guideline considers comorbid chronic pain only in the section of medical treatment.

Getting back to the before mentioned specific mechanism-based treatment approach, even if trauma-focused psychotherapy has been shown to be effective in the treatment of psychological trauma, the efficacy of trauma-focused psychotherapy in patients with chronic pain and previous psychological trauma as well as the underlying mechanisms have not yet been investigated in depth so far. However, there are some hints that this might be a promising approach [30-33]. The same is true for treatment of pain. It is not known whether such common treatment approaches are effective in patients with comorbid psychological trauma. This is important because research shows that patients with anxiety disorders and high comorbid pain interference have a lower likelihood of responding to treatment than patients without pain interference [34]. Therefore, research in regard to treatment of chronic pain with comorbid psychological trauma is necessary. Moreover, recent studies indicate that trauma-focused treatment approaches can successfully reduce comorbid symptoms also in non-posttraumatic stress symptomatology in which trauma is judged to be of causative significance [35].

A psychological first line treatment for psychological trauma, that has been proven to be efficacious, is eye movement desensitization and reprocessing (EMDR) [25,28,36-38]. Although EMDR was originally developed for individuals who had experienced psychological trauma, the neurobiological similarities found in patients who suffer from posttraumatic stress symptoms and chronic pain disorders encouraged scientists to explore the utilization of EMDR in the treatment of chronic pain, even in the absence of psychological trauma. Accordingly, there is an increasing amount of literature regarding the use of EMDR in the treatment of chronic pain.

Several studies report that EMDR is able to reduce pain intensity or even eliminate pain completely in patients with different pain disorders (e.g., phantom limb pain, fibromyalgia, and headache) [39]. Furthermore, EMDR has been found to improve pain coping abilities and to facilitate relaxation abilities that finally results in the reduction of pain and pain-related attitudes and beliefs [40].

However, until now there have predominantly been case-reports and case-series and none of them focused on CBP. Moreover, though there is first evidence that EMDR is effective and useful for the treatment of chronic pain, the underlying mechanisms still remain unclear.

Accordingly, we designed a randomized controlled trial on change in pain intensity by EMDR-treatment in patients suffering from chronic non-specific back pain and previous psychological trauma. In addition, our core study is expanded by a comprehensive neurobiological and psychosocial assessment to explore potential underlying mechanisms.

Therefore, the purpose of the present study is twofold: 

1.) To determine whether a 10-session standardized EMDR short-term intervention in addition to treatment as usual (TAU) reduces pain intensity in CBP patients with experienced psychological trauma vs. TAU alone, and

2.) to explore additionally potential underlying mechanisms.

## Methods and design

This study is part of the consortium ‘Localized and Generalized Musculoskeletal Pain: Psychobiological Mechanisms and Implications for Treatment’ [41] funded by the German Federal Ministry of Research and Education (01EC1010A-F). More details concerning LOGIN can be found elsewhere [41-43]. This report is part of subproject number six (SP6; 01EC1010A) ‘Subgroups Characterised by Psychological Trauma, Mental Co-morbidity, and Psychobiological Patterns and Their Specialised Treatment’ [43]. This trial has received ethics approval from the Ethics Committee Heidelberg (approval No. S-261/2010), and is registered within clinical trails.gov (NCT01850875). All participants must provide written informed consent.

### Design

**Figure 1:**
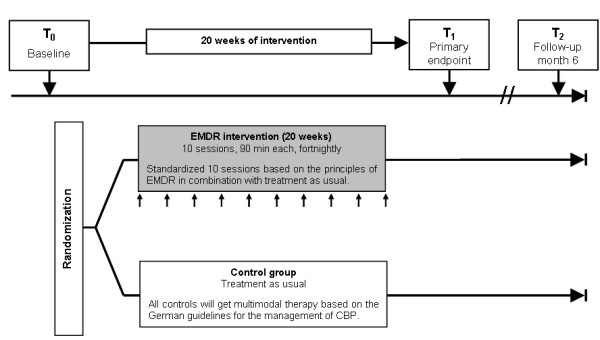
**Intervention scheme.** Outcome assessments will be done before randomization (T_0_, baseline), two weeks after intervention/treatment as usual (T_1_, primary endpoint), and six months after intervention/treatment as usual (T_2_, follow-up). CBP: non-specific chronic back pain.

The study is designed as a randomized controlled trial with two parallel arms (see Figure 1). Forty patients will be randomized to either the treatment group (n = 20) or the control group (n = 20). The treatment group will receive EMDR in addition to TAU. The control group will receive TAU alone.

### Participants

Participants will be recruited from the southwestern district “Rhein-Neckar” of Germany, through subproject six [43] of the LOGIN consortium. Patients will be offered enrolment, when in addition to CBP they additionally fulfill the criteria for experienced psychological trauma (see Table 1). Recruitment will be done consecutively until a sample size of n = 40 patients is reached (control group: n = 20; intervention group: n = 20). Randomization will be done centrally with the established randomization program RANDI_2_[44,45] after patient inclusion by an independent person not involved in treatment or assessment. Allocation concealment will be ensured, as this person will not release the randomization code until the patient has been recruited into the trial, which takes place after all baseline assessments have been completed.

**Table 1:** Inclusion and exclusion criteria

**Inclusion criteria**	
**Diagnosis**	1.) Non-specific CBP ≥ 45 days during the past three months, and
	2.) History of experienced psychological trauma*
**Age**	At least 18 years of age
**Language**	Fluent German language skills
**Exclusion criteria**	
**Pathology**	- Specific pathologies of CBP (e.g., spinal canal stenosis, disc hernia, spondylolisthesis, infection, malignancy, Bechterew disease, and fracture)
	- Sciatica pain ≥ than back pain
	- Severe physical or psychiatric co-morbidity
	- Cognitive impairment
**Pension status**	Active or planned worker’s compensation, disability or personal injury claims
**Medical status**	Planned other or ongoing psychotherapy, change in medication in the last three months

*Psychological trauma will be assessed using the German Version of the Posttraumatic Diagnostic Scale (PDS-D)[46]*. Patients who at least answer one trauma item positive will be classified as patients with experienced psychological trauma. All other patients will be classified as patients without psychological trauma. For details see*[43]*.*

Outcome assessments will be conducted by an assessor blind to treatment allocation. An employee outside the research team will feed data into the computer in separate datasheets so that the researchers can analyze data without having access to information about the allocation.

### Inclusion and exclusion criteria

Diagnosis of non-specific chronic back pain and psychological trauma is validated within LOGIN subproject six. For details see [43]. Briefly, patients must report more than 44 days of low back pain within at least the last three months (=CBP). Then, to verify inclusion and exclusion criteria (Table 1), all participants will be questioned about their past medical history and about co-morbidities. In addition, patients will also receive a physical examination (including blood tests and if indicated further technical investigations such as x-ray or MRI) that attaches special importance to findings which indicate a specific origin of back pain. Therefore the “red flags” (hints for the presence of serious pathology according to the Agency for Health Care Policy and Research Low Back Guidelines [47]) will be considered and former medical reports and discharge letters will be taken into account whenever available. In case of signs for serious pathological findings, participants will be excluded and a further investigation will be advised.

Psychological trauma will be diagnosed with the German Version of the Posttraumatic Distress Scale (PDS-D) [46]. Patients who at least answer one trauma item positive will be classified as patients with experienced psychological trauma. All other patients will be classified as patients without psychological trauma. For details see [43].

Participants that meet all the inclusion criteria will have the trial explained to them in detail and then be asked to provide written informed consent before being enrolled into the trial.

### Description of intervention

All the participants will continue to receive their usual routine care (e.g., general support and advice, physiotherapy, simple analgesics for their symptoms) from their own general practitioners and other healthcare providers in accordance with the guidelines for the treatment of CBP in Germany [19].

In addition to TAU, the participants assigned to the EMDR intervention group will receive a manualized outpatient intervention that is based on the principles of EMDR-trauma therapy [48,49] and incorporates standardized EMDR pain protocols [50,51]. The participants will receive ten 90-minute sessions in an individual setting. The intervention manual will be prepared in cooperation with an expert in trauma therapy. The EMDR treatment will be provided by EMDR-trained psychologists and physicians (who have participated in an EMDRIA-approved Basic Training program). The therapists will be trained in advance specifically in the use of EMDR for chronic pain patients and will be supervised regularly by an EMDRIA-approved consultant. All the sessions will be video-recorded. The therapists’ treatment fidelity will be managed by using standardized treatment protocols and regular supervision, including monitoring the video-recorded sessions.

Based on the theoretical rationale proposed by Carlson and colleagues [52] and the general recommendations for evaluating empirical EMDR studies [53], a ‘dose’ of ten 90-minute sessions given over a 20-week period was chosen to guarantee sufficient treatment length. Although there is currently no definitive agreement on the number of EMDR treatment sessions required [48] and the existent literature suggests that EMDR is effective with fewer than ten sessions (e.g., van Rood 2009 [54]), we postulate that this number of treatment sessions will allow adequate time for testing comparative treatments as well.

Based on the theoretical rationale proposed by Carlson and colleagues [52], and the general recommendations for the evaluation of empirical EMDR studies [53] a ‘dose’ of ten sessions à 90 minutes per session given over a 20-weeks period is chosen to guarantee sufficient treatment length. Even though there is currently no definitive agreement on the number of EMDR treatment sessions required [48], and existent literature supports EMDR effectiveness in fewer than ten sessions (e.g., van Rood 2009 [54]), we postulate that this number of treatment sessions would allow adequate time to test comparative treatments as well.

### Data acquisition

All the outcome assessments and the EMDR sessions will be conducted at the Department of General Internal Medicine and Psychosomatics, University Hospital Heidelberg, Germany. The outcome assessments will be performed by an independent evaluator who is blinded to the treatment allocation at three time points: baseline (T_0_, before randomization), two weeks after the intervention/TAU (T_1_, primary endpoint), and six months after the intervention/TAU (T_2_, follow-up). The physical examination and the clinical interviews will only be conducted at baseline. The assessor will be trained at the beginning of the study and will be observed regularly to ensure the reliability and validity of the measures.

### Outcome measures

The primary endpoint with respect to the efficacy of the standardized short-term EMDR intervention for CBP is the change in the intensity of back pain from T_0_ to T_1_ measured on a numeric rating scale (NRS 0–10, averaged over four weeks). The NRS for pain intensity has proven to be a reliable and valid instrument for assessing treatment effects in pain trials [55].

The secondary outcomes, which include distinct pain variables (mean pain duration, pain experience, and pain intensity at T_2_), medication intake, health-related quality of life (SF-12), anxiety and depression (HADS), dissociation symptoms (FDS-20), psychological resilience (RS-11), and somatization (SCL-90R subscale somatization), will be measured at all assessment points (T_1_ and T_2_).

In addition to the efficacy endpoints, we assess the peripheral and central modulation of pain (quantitative sensory testing, QST, and conditioned pain modulation, CPM) in all the patients to explore the differences in pain processing induced by the EMDR intervention. An overview of the primary and secondary outcomes is shown in Table 2.

**Table 2:** Outcome measures

**Primary outcome**	
**Pain intensity**	• *Numerical rating scale (NRS)*, ranging from 0 ‘no pain’ to 10 ‘worst pain imaginable’ [55].
**Secondary outcomes**	
**Pain dimensions**	• *Pain duration* (number of painful days in the last 4 weeks).
	• *Pain Experience Scale (SES)* measures the affective and sensory dimensions of pain [56].
**Medication**	• Change in medication intake.
**Disability**	• *12-Item Short Form Health Survey (SF-12)* measures health-related quality of life [57,58].
**Depression and Anxiety**	• *Hospital Anxiety and Depression Scale (HADS-D)*[59].
**Stress/ Trauma**	• *Change in Posttraumatic Diagnostic Scale levels (PDS)*[60]*.*
	• *Dissociation symptoms questionnaire (DES/FDS-20)* for assessment of dissociative symptoms [61].
**Somatization**	• *Somatization scores* of the SCL-90R subscale somatization (SCL-90R) [62].
**Somato-sensory function**	• *Quantitative Sensory Testing (QST)* for assessment of all relevant aspects of the somatosensory system including large and small fiber function as well as signs of central sensitization (dynamic tactile allodynia, punctate mechanical hyperalgesia) [63].
**Conditioned pain modulation**	*• Conditioned Pain Modulation (CPM)* paradigm for assessment of inhibitory pain modulating mechanisms [64]. Difference in pressure pain threshold before and after inducing CPM by a phasic heat pain stimulus will be measured.

Moreover, as part of the LOGIN study, all the participants in our study will be assessed for a shared core set of variables, including a comprehensive assessment of neuromodulators (plasma nerve-growth-factor, plasma endocannabinoid and lipid levels) and the functional and structural changes in functional neuroimaging (fMRI), before and after treatment (for further details, see [43]).

### Assessment of safety

Serious adverse events (SAEs) will be documented and reported casuistically.

### Sample size estimation

The sample size calculation is based on the primary efficacy endpoint: change in the pain intensity (numeric rating scale 0–10) after the intervention (T_1_). The expected effect size for our trial is derived from previous evidence about the effect of EMDR therapy for chronic pain patients.

The two controlled trials currently available are characterized by high effect sizes with Hedges’ g ranging from −1.12 [65] to −6.87 [66]. Based on this data, an effect size of at least 1.0 can be expected after ten sessions of treatment. A sample size of 17 patients in each group will have 80% power to detect an effect size of 1.0 using a t-test for independent groups with a 0.05 two-sided significance level. Based on comparable studies and our own experience with pain trials, we expect a 20% drop-out rate. Therefore, a total sample size of 40 patients is required for the study. The patients who are lost to follow up and/or non-compliant patients will be handled using an intention-to-treat procedure.

### Statistical analyses

The data analysis will follow the evaluation standards for experimental study designs with control groups, using descriptive methods as well as inferential statistics. The data will be analyzed using an intention-to-treat approach. Because of the small sample size in the study, multiple imputation (the gold standard for handling missing values) is not appropriate. Therefore, only complete cases will be used in the final analysis. Dropouts, withdrawals and missing values will be analyzed in detail; different techniques will be used to assess them, and these techniques will be compared using sensitivity analyses. Descriptive statistics will be presented as the means and standard deviations for the continuous variables and absolute numbers and percentages for the categorical variables. The questionnaires will be processed in accordance with questionnaire manuals.

The differences between the change scores of the patient groups will be analyzed using independent t-tests. Moreover, potential negative effects will be reported descriptively (e.g., pain intensity after the trauma-focused intervention > pain intensity before the trauma focused intervention). Preliminary effect sizes will be estimated using Cohen’s d.

Pre-processing analyses and statistical analyses of quantitative sensory testing data will be performed according to the protocol used by Rolke et al. [63]. To quantify conditioned pain modulation, the pressure pain threshold before a phasic heat stimulus will be subtracted from the pressure pain threshold after the phasic heat stimulus. A negative value indicates an analgesic effect due to CPM [64].

## Discussion

Although CBP is often accompanied by psychological trauma and posttraumatic stress symptoms, a specific trauma-focused approach for this subgroup of CBP cases has been a neglected issue to date. Eye movement desensitization and reprocessing, originally developed as a short-duration, high-efficiency treatment approach for posttraumatic stress symptoms, is a promising approach for treating CBP in patients who have experienced psychological trauma. The preliminary evidence suggests that EMDR treatment may benefit patients with medically unexplained somatic symptoms [54]. To date, several uncontrolled studies using EMDR have been conducted for a multitude of various pain syndromes [40,66-70]. These studies suggest that EMDR may be a safe and promising treatment option for chronic pain conditions.

However, most of the research conducted thus far has been preliminary in nature and characterized by small sample sizes, heterogeneous patient groups, the lack of randomization and the absence of adequate control groups. Moreover, to our knowledge, none of these studies have evaluated the efficacy of EMDR in patients suffering CBP. Thus, there is an urgent need for further exploration of the efficacy of EMDR for treating chronic pain patients using well-designed studies with standardized EMDR interventions of sufficient treatment length and adequate control groups. Accordingly, we designed this randomized controlled trial on the effects of EMDR treatment for non-specific chronic back pain patients who have experienced psychological trauma to determine whether a standardized short-term EMDR intervention reduces pain intensity in this subgroup of CBP patients. Because this is the first randomized controlled trial on this topic, the results of this study will encourage a more specific trauma-focused treatment approach, especially for the pain patients who have experienced psychological trauma.

However, although there is preliminary evidence that EMDR is effective and useful for the treatment of chronic pain, the underlying mechanisms remain unclear. Therefore, our core study will be supplemented by a comprehensive neurophysiologic and psychosocial assessment to explore these mechanisms of action.

Notably, the study is part of the LOGIN consortium study [41]. LOGIN comprises seven subprojects, including basic and applied research as well as preclinical and clinical projects utilizing a wide variety of research methods. As part of this consortium study, all the participants in our study will additionally be assessed for a shared core set of variables to investigate similar pathogenetic mechanisms. This approach enables us to expand our exploration of the underlying mechanisms by conducting a comprehensive assessment of systemic neuromodulators (plasma nerve-growth-factor, plasma endocannabinoid and lipid levels) and the functional and structural changes with functional neuroimaging (fMRI) before and after treatment and to explore the differences in pain processing induced by the EMDR intervention. This approach will be supported by the translational aspects of LOGIN. Thus, using the synergy of the different subprojects, the timely translation, implementation and dissemination of the results will be guaranteed.

The findings of this study might contribute to improvements in the treatment of patients suffering from CBP as well as to better understanding of the mechanisms of action underlying EMDR in chronic pain patients.

## Competing interest

The authors declare that they have no competing interests.

## Authors’ contributions

AG and JT have made substantial contributions to conception and design and have been involved in drafting the manuscript. MH and WE have made substantial contributions to conception and design and revised the manuscript critically for important intellectual content. SL, SJ, GHS were involved in data collection and revised the manuscript critically for important intellectual content. All authors read and approved the final version of the manuscript to be published.

## Pre-publication history

The pre-publication history for this paper can be accessed here:

http://www.biomedcentral.com/1471-2474/14/256/prepub
